# Gene expression profiling of idiopathic interstitial pneumonias (IIPs): identification of potential diagnostic markers and therapeutic targets

**DOI:** 10.1186/s12881-017-0449-9

**Published:** 2017-08-18

**Authors:** Yasushi Horimasu, Nobuhisa Ishikawa, Masaya Taniwaki, Kakuhiro Yamaguchi, Kosuke Hamai, Hiroshi Iwamoto, Shinichiro Ohshimo, Hironobu Hamada, Noboru Hattori, Morihito Okada, Koji Arihiro, Yuji Ohtsuki, Nobuoki Kohno

**Affiliations:** 1 Department of Molecular and Internal Medicine, Hiroshima University, 1-2-3 Kasumi, Minami-ku, Hiroshima, 734-8551 Japan; 2 Department of Physical Analysis and Therapeutic Sciences, Hiroshima University, 1-2-3 Kasumi, Minami-ku, Hiroshima, 734-8551 Japan; 3 Department of Surgical Oncology, Graduate School of Biomedical and Health Sciences, Hiroshima University, 1-2-3 Kasumi, Minami-ku, Hiroshima, 734-8551 Japan; 4 Department of Anatomical Pathology, Hiroshima University Hospital, 1-2-3 Kasumi, Minami-ku, Hiroshima, 734-8551 Japan; 5 Division of Pathology, Matsuyama-shimin Hospital, 2-6-5 Ohtemachi, Matsuyama, 790-0067 Japan; 6 Department of Respiratory Medicine, Hiroshima Prefectural Hospital, 1-5-54 Ujina-Kanda, Minami-ku, Hiroshima, 734-8530 Japan

**Keywords:** Biomarker, Desmoglein 3, Gene expression profiles, Molecular targeted therapy, Pathway analysis, Pulmonary fibrosis

## Abstract

**Background:**

Chronic fibrosing idiopathic interstitial pneumonia (IIP) is characterized by alveolar epithelial damage, activation of fibroblast proliferation, and loss of normal pulmonary architecture and function. This study aims to investigate the genetic backgrounds of IIP through gene expression profiling and pathway analysis, and to identify potential biomarkers that can aid in diagnosis and serve as novel therapeutic targets.

**Methods:**

RNA extracted from lung specimens of 12 patients with chronic fibrosing IIP was profiled using Illumina Human WG-6 v3 BeadChips, and Ingenuity Pathway Analysis was performed to identify altered functional and canonical signaling pathways. For validating the results from gene expression analysis, immunohistochemical staining of 10 patients with chronic fibrosing IIP was performed.

**Results:**

Ninety-eight genes were upregulated in IIP patients relative to control subjects. Some of the upregulated genes, namely desmoglein 3 (*DSG3*), protocadherin gamma-A9 (*PCDHGA9*) and discoidin domain-containing receptor 1 (*DDR1*) are implicated in cell-cell interaction and/or adhesion; some, namely collagen type VII, alpha 1 (*COL7A1*), contactin-associated protein-like 3B (*CNTNAP3B*) and mucin-1 (*MUC1*) are encoding the extracellular matrix molecule or the molecules involved in cell-matrix interactions; and the others, namely *CDC25C* and growth factor independent protein 1B (*GFI1B*) are known to affect cell proliferation by affecting the progression of cell cycle or regulating transcription. According to pathway analysis, alternated pathways in IIP were related to cell death and survival and cellular growth and proliferation, which are more similar to cancer than to inflammatory response and immunological diseases. Using immunohistochemistry, we further validate that *DSG3*, the most highly upregulated gene, shows higher expression in chronic fibrosing IIP lung as compared to control lung.

**Conclusion:**

We identified several genes upregulated in chronic fibrosing IIP patients as compared to control, and found genes and pathways implicated in cancer, rather than in inflammatory or immunological disease to play important roles in the pathogenesis of IIPs. Moreover, *DSG3* is a novel potential biomarker for chronic fibrosing IIP with its significantly high expression in IIP lung.

## Background

Idiopathic interstitial pneumonia (IIP) encompasses a group of diffuse parenchymal lung diseases characterized by interstitial involvement resulting from various patterns of inflammation and fibrosis of unknown cause. Based on histological features, IIP has been further classified into several subtypes, including idiopathic pulmonary fibrosis (IPF), which has the hallmark histopathologic feature described as usual interstitial pneumonia and nonspecific interstitial pneumonia (NSIP) [1–3]. The latest statement from American Thoracic Society (ATS) and European Respiratory Society (ERS) proposed the category “chronic fibrosing IIP” encompassing both IPF and NSIP [3], because separation between these two diseases is difficult, with significant clinical, radiological, and pathological overlap between them [4].

IPF is one of the most common and aggressive types of IIP and is characterized by alveolar epithelial damage that leads to inadequate tissue repair, collagen accumulation, and fibroblast proliferation, although the underlying molecular mechanisms remain unclear [5]. Over the last decade, the few therapeutic options available have not been very effective and the outcome of IPF patients is poor [6]. Pirfenidone is the first anti-fibrotic agent to be approved for IPF treatment, with its efficacy and tolerability supported by several clinical trials and surveillance [7–10]. Recently, nintedanib, a multiple tyrosine kinase inhibitor, also demonstrated clinical efficacy for IPF patients [11]. However, these drugs only reduce the decline in forced vital capacity, without halting disease progression in all patients. Therefore, new diagnostic tools and therapeutic strategies, including molecular targeting drugs, are urgently needed.

Systematic analysis of the expression level of thousands of genes using microarray is an effective approach for identifying molecules that are altered in pulmonary fibrosis or after treatment with anti-fibrotic agents [12]. Our group, as well as others, have performed high-throughput screens combined with gene expression analysis of lung diseases including cancers, and identified various potential targets for the development of new diagnostic tools and therapies [13–16]. However, few such analyses have been performed for IIP [17–21], and most of these studies have been performed in Caucasian populations with very limited data available from Japanese populations.

The present study aims to delineate the molecular mechanisms of pulmonary fibrosis and identify potential disease-specific biomarkers and/or therapeutic targets in the chronic fibrosing IIPs patients by using genome-wide microarray analysis followed by canonical pathway analysis.

## Methods

### Patients and clinical samples

Tissue samples were obtained by surgical lung biopsy from Japanese patients with newly diagnosed IIP at the Hiroshima University Hospital (Hiroshima, Japan) and who have never taken medication for IIPs before. All surgical lung specimens were immediately frozen and stored at −80 °C for later analysis. Each patient underwent physical examination, pulmonary function tests, high-resolution computed tomography, bronchoscopy, and bronchoalveolar lavage. IPF and NSIP were diagnosed according to the ATS/ERS criteria published in 2002 [22]. Patients with evidence of collagen vascular disease, chronic hypersensitivity pneumonia, and other known causes of interstitial lung diseases (ILDs) were excluded. Control lung specimens for microarray analysis consisted of total RNA from three lungs (Caucasians aged 32–61 years; cause of death: sudden death) purchased from BD Biosciences Clontech (Lot Number 7080277; Palo Alto, CA, USA). Control tissues for immunohistochemistry were obtained from the healthy areas of lungs, removed locally, along with lung tumors. This study was approved by the Ethics Committee of Hiroshima University Hospital (IRB M33 and 326) and conducted in accordance with ethical standards established in the Helsinki Declaration of 1975. All participants provided written informed consent for the use of tissue specimens for the study and the publication of their individual data. Clinical characteristics of the 12 IIP patients are summarized in Table 1.

**Table 1:** Clinical characteristics of idiopathic interstitial pneumonia cases

Variables	Microarray cohort	IHC cohort
	(*n* = 12)	(*n* = 10)
Age (years)	66.2 ± 2.1	63.1 ± 1.9
Gender (male/female)*	9/3	7/3
Smoking history (Smoker/non-smoker) *	9/3	7/3
Pack-years*	23.8 ± 6.9	26.4 ± 7.8
Disease category (IPF/ NSIP)	7/5	5/5
Pulmonary function test		
VC (% predicted)	86.0 ± 6.2	88.9 ± 6.6
DLco (% predicted)	57.2 ± 5.3	54.1 ± 5.5

Values are expressed as mean ± SEM or as a number

*P < 0.05 (Mann-Whitney U or χ^2^ test between two groups)

*IHC* immunohistochemical analysis, *IPF* idiopathic pulmonary fibrosis, *NSIP* nonspecific interstitial pneumonia, *VC* vital capacity, *DL*_*CO*_ single-breath carbon monoxide diffusing capacity

### RNA isolation and gene expression profiling

Gene expression profiles of frozen tissue specimens from 12 IIP patients, derived from the central part of the surgical lung biopsy, were analyzed by GP Biosciences Ltd. (Kanagawa, Japan). RNA quality was verified using the RNA6000 Nano Assay on an Agilent 2100 Bioanalyzer (Agilent Technologies, Santa Clara, CA, USA). Illumina Human WG-6 v3 BeadArrays (Illumina Inc., San Diego, CA, USA) with about 48,000 transcripts were used according to the manufacturer’s instructions. An Illumina TotalPrep RNA amplification kit (Ambion, Inc., Austin, TX, USA) was used to obtain biotin-labeled cRNA from 500 ng total RNA. As a control probe, normal human lung poly(A) RNA (BD Biosciences Clontech) was amplified under the same conditions. cRNA was synthesized overnight (18 h), labeled, and hybridized to the chip at 58 °C overnight. Hybridized arrays were labeled with streptavidin-Cy3 (PA43001; Amersham, Buckinghamshire, UK) and scanned with an Illumina BeadArray reader (Illumina Inc.). Scanned images were imported into BeadStudio v3 software (Illumina Inc.) for extraction, quality adjustment, and quantile normalization. Satisfactory quality was observed for all arrays and samples.

### Functional and canonical pathway analyses

The microarray gene expression data was analyzed using Ingenuity Pathway Analysis (IPA; Ingenuity Systems, Redwood City, CA, USA) to determine whether genes associated with particular diseases, biological functions, or canonical signaling pathways were preferentially up- or downregulated in IIP patients relative to control subjects. Diseases and biological functions for which differential gene expression was observed were grouped into three categories: (1) diseases and disorders; (2) molecular and cellular functions; and (3) physiological system development and function.

### Clustering analysis of microarray data

To assess the difference and similarity in the gene expression profile between IPF and NSIP, a hierarchical clustering method was applied to genes and IIP subtypes. To obtain reproducible clusters for classifying the 12 IIP patients, 159 genes were selected for which valid data was obtained in 80% of the experiments, and whose expression ratios varied by standard deviations of >3.0. Gene Cluster 3.0 and Java TreeView software developed by Eisen et al. were used to analyze the data [23, 24]. Before applying the clustering algorithm, the fluorescence ratio for each spot was log-transformed and the data for each sample was median-centered to remove experimental biases.

### Immunohistochemical staining and morphometric analysis

To evaluate the protein expression of two upregulated genes, desmoglein 3 (*DSG3*) and Krebs von den lungen-6 (*KL-6*)/Mucin 1 (*MUC1*), clinical tissue sections from 5 IPF patients, 5 NSIP patients, and 5 control lungs were stained using ENVISION+ Kit/horseradish peroxidase (HRP) (Dako Japan, Tokyo, Japan), as previously described [25]. For antigen retrieval, slides were immersed in Target Retrieval Solution, Citrate pH 6 (Dako Japan) and boiled at 108 °C for 15 min in an autoclave. After blocking endogenous peroxidase activity with 0.03% H_2_O_2_ for 30 min, sections were incubated with mouse anti-human *DSG3* (Clone #216519; R&D Systems, Minneapolis, MN, USA) and KL-6 antibodies, which were purified as previously described [26]. The slides were then treated with HRP-labeled anti-mouse IgG secondary antibody followed by the addition of a chromogenic substrate. Sections were counterstained with hematoxylin. Image-Pro Plus 6.3 (Media Cybernetics. Inc. Rockville, MD, USA) was used for morphometric analysis to quantify the positively stained areas in the lung tissue, as previously described [27].

### Statistical analyses

Data were analyzed with SPSS for Windows, version 18.0 (SPSS Inc. Chicago, IL, USA) and are presented as mean ± SEM. Data for individual variables from the various groups were analyzed by the Kruskal-Wallis test followed by multiple comparisons using rank sums [28]. Mean differences were considered statistically significant at *P* < 0.05.

## Results

### Identification of genes up−/downregulated in IIP

Clinical characteristics of the 12 patients with chronic fibrosing IIP (IPF, *n* = 7; NSIP, *n* = 5) analyzed by microarray were shown in Table 1. In total, 98 genes were upregulated while 1193 were downregulated in the lung tissue of IIP patients compared to control subjects, based on expression ratios that were >20.0 or <0.05 respectively, in at least 75% (i.e., 9 out of 12) of informative cases. The top 50 genes upregulated in IIP are listed in Table 2. Some of the upregulated genes, namely *DSG3*, protocadherin gamma-A9 (*PCDHGA9*) and discoidin domain-containing receptor 1 (*DDR1*) are implicated in cell-cell interaction and/or adhesion; some, namely collagen type VII, alpha 1 (*COL7A1*), contactin-associated protein-like 3B (*CNTNAP3B*) and *MUC1* are encoding the extracellular matrix molecule or the molecules involved in cell-matrix interactions; and the others, namely *CDC25C* and growth factor independent protein 1B (*GFI1B*) are known to affect cell proliferation by affecting the progression of cell cycle or regulating transcription. Of these, DDR1 and KL-6/MUC1 have been previously reported as biomarkers for ILD [29, 30]. On the other hand, the top 50 genes downregulated in IIP are listed in Table 3. Some of these, namely Defensin alpha 1 (*DEFA1*), *DEFA3* and Mucin 7 (*MUC7*) are known to play important roles in antimicrobial defense system in upper respiratory tract. Additionally, we also found that interleukin 10 (*IL-10*), which is known to be one of the inhibitor of Th1 cells was significantly downregulated.

**Table 2:** Top 50 genes upregulated in idiopathic interstitial pneumonia (IIP) patients

Gene symbol	Gene name	Fold change Fibrotic IIP/control
DSG3	Desmoglein 3	321.2
KLRD1	Killer cell lectin-like receptor subfamily D, member 1	252.8
OTUB1	OTU domain, ubiquitin aldehyde binding 1	246.7
ZFP92	ZFP92 zinc finger protein	222.9
NLGN4Y	Neuroligin 4, Y-linked	217.2
GLB1L3	Galactosidase, beta 1-like 3	214.8
CDC25C	Cell division cycle 25C	211.4
CNTNAP3B	Contactin-associated protein-like 3B	186.3
SRGAP2	SLIT-ROBO Rho GTPase-activating protein 2	174.5
NEK3	NIMA-related kinase 3	167.0
SYT8	Synaptotagmin VIII	156.9
TLR10	Toll-like receptor 10	145.6
DST	Dystonin	140.3
COL7A1	Collagen type VII, alpha 1	139.4
UTY	Ubiquitously transcribed tetratricopeptide repeat-containing, Y-linked	127.8
CYorf15A	Chromosome Y open reading frame 15A	121.8
PCDHGA9	Protocadherin gamma subfamily A, 9	120.9
OXTR	Oxytocin receptor	111.8
AIM2	Absent in melanoma 2	107.5
TMSB4Y	Thymosin beta 4, Y-linked	106.4
YBEY	ybeY metallopeptidase	104.4
UBE2D2	Ubiquitin-conjugating enzyme E2D 2	102.7
DDR1	Discoidin domain receptor tyrosine kinase 1	99.2
ZFY	Zinc finger protein, Y-linked	98.0
SLC17A9	Solute carrier family 17, member 9	98.0
EIF1AY	Eukaryotic translation initiation factor 1A, Y-linked	96.8
ANO9	Anoctamin 9	91.6
DDX3Y	DEAD (Asp-Glu-Ala-Asp) box polypeptide 3, Y-linked	88.7
HBS1L	HBS1-like (*S. cerevisiae*)	88.6
MSL3	Male-specific lethal 3 homolog (*D. melanogaster*)	85.2
MUC1	Mucin 1, cell surface-associated	83.3
PLCH2	Phospholipase C, eta 2	82.5
LPGAT1	Lysophosphatidylglycerol acyltransferase 1	81.8
DDX6	DEAD (Asp-Glu-Ala-Asp) box helicase 6	80.4
SDK2	sidekick cell adhesion molecule 2	79.2
C1QTNF1	C1q and tumor necrosis factor related protein 1	78.8
ZRANB2	zinc finger, RAN-binding domain containing 2	78.6
ZNF300	zinc finger protein 300	76.9
DMBT1	deleted in malignant brain tumors 1	76.7
GFI1B	growth factor independent 1B transcription repressor	75.1
SLFN13	schlafen family member 13	72.1
CDK5RAP3	CDK5 regulatory subunit associated protein 3	70.8
SLCO3A1	solute carrier organic anion transporter family, member 3A1	70.5
SNRNP70	small nuclear ribonucleoprotein 70 kDa (U1)	66.2
IL18BP	interleukin 18 binding protein	64.7
PCNXL2	pecanex-like 2 (Drosophila)	64.6
ZSCAN29	zinc finger and SCAN domain containing 29	63.7
ESR2	estrogen receptor 2 (ER beta)	62.5
FBXO41	F-box protein 41	61.7
MRPL20	mitochondrial ribosomal protein L20	61.3

**Table 3:** Top 50 known genes downregulated in IIP patients

Gene symbol	Gene name	Fold change Fibrotic IIP/control
PRB2	Proline rich protein BstNI subfamily 2	8.35E-04
DEFA3	Defensin alpha 3	9.05E-04
DEFA1	Defensin alpha 1	1.09E-03
PRB1	Proline rich protein BstNI subfamily 1	1.21E-03
HLA-DRB5	Major histocompatibility complex, class II, DR beta 5	1.38E-03
CYP1A2	Cytochrome P450 family 1 subfamily A member 2	1.59E-03
VNN2	Vanin 2	1.73E-03
MUC7	Mucin 7	1.82E-03
PRB3	Proline rich protein BstNI subfamily 3	1.85E-03
FPR2	Formyl peptide receptor 2	1.88E-03
WDR47	WD repeat domain 47	1.90E-03
HLA-C	Major histocompatibility complex, class I, C	1.91E-03
HIST1H2BD	Histone cluster 1 H2B family member d	1.94E-03
CCRL1	Chemokine (C-C motif) receptor-like 1	2.00E-03
UMOD	Uromodulin	2.09E-03
HIST1H1E	Histone cluster 1 H1 family member e	2.11E-03
HIST1H2AE	Histone cluster 1 H2A family member e	2.19E-03
FCAR	Fc fragment of IgA receptor	2.24E-03
SLCO1A2	Solute carrier organic anion transporter family member 1A2	2.26E-03
RNASE3	Ribonuclease A family member 3	2.27E-03
WWP2	WW domain containing E3 ubiquitin protein ligase 2	2.33E-03
BPIFB2	BPI fold containing family B member 2	2.34E-03
LPO	Lactoperoxidase	2.40E-03
ITLN1	Intelectin 1	2.42E-03
NAIP	NLR family apoptosis inhibitory protein	2.42E-03
CCDC85A	Coiled-coil domain containing 85A	2.46E-03
MAP4	Microtubule associated protein 4	2.50E-03
PRH2	Proline rich protein HaeIII subfamily 2	2.54E-03
IL10	Interleukin 10	2.59E-03
CELA2A	Chymotrypsin like elastase family member 2A	2.63E-03
RGPD1	RANBP2-like and GRIP domain containing 1	2.64E-03
LAT2	Linker for activation of T-cells family member 2	2.66E-03
LRCH1	Leucine rich repeats and calponin homology domain containing 1	2.67E-03
HFE	Hemochromatosis	2.79E-03
GPM6A	Glycoprotein M6A	2.81E-03
APOL1	Apolipoprotein L1	2.84E-03
FCGR3B	Fc fragment of IgG receptor IIIb	2.89E-03
S100A12	S100 calcium binding protein A12	2.92E-03
CCK	Cholecystokinin	2.93E-03
FCRL6	Fc receptor like 6	2.94E-03
OPRPN	Opiorphin prepropeptide	2.96E-03
HMGCS2	3-hydroxy-3-methylglutaryl-CoA synthase 2	2.98E-03
C8orf37	chromosome 8 open reading frame 37	3.02E-03
TCN1	Transcobalamin 1	3.06E-03
HIST1H4I	Histone cluster 1 H4 family member i	3.11E-03
SLC22A12	Solute carrier family 22 member 12	3.14E-03
RGPD5	RANBP2-like and GRIP domain containing 5	3.26E-03
PRTG	Protogenin	3.32E-03
C8B	Complement C8 beta chain	3.38E-03
CLEC4M	C-type lectin domain family 4 member M	3.46E-03

### Functional and canonical pathway analyses

As shown in Table 4, IPA software revealed that the most highly-altered entry in IIP patients relative to control subjects for (1) diseases and disorders; (2) molecular and cellular functions; and (3) physiological system development and function was cancer, cellular movement, and cardiovascular system respectively. The top five canonical signaling pathways associated with the genes were antigen presentation pathway, cytotoxic T lymphocyte-mediated apoptosis of target cells, dendritic cell maturation, molecular mechanisms of cancer, and crosstalk between dendritic cells and natural killer cells. Thus, a number of genes and pathways related to cancer, cell death and survival, and cellular growth and proliferation were differentially expressed in IIP patients.

**Table 4:** Top 5 functional and canonical pathways from the Ingenuity Pathway Analysis

Functional analysis/name	*P* value	Number of molecules
Diseases and disorders		
Cancer	2.47E − 09–4.63E − 03	770
Renal and urological disease	3.66E − 08–3.83E − 03	99
Inflammatory response	6.96E − 08–4.28E − 03	263
Immunological disease	3.91E − 07–3.15E − 03	219
Hematological disease	4.54E − 07–4.41E − 03	141
Molecular and cellular functions		
Cellular movement	5.28E − 11–4.43E − 03	295
Cell death and survival	2.11E − 07–4.72E − 03	437
Cell morphology	8.16E − 07–4.94E − 03	105
Cellular growth and proliferation	1.51E − 06–4.17E − 03	423
Cellular development	2.62E − 06–4.92E − 03	372
Physiological system development and function		
Cardiovascular system development and function	1.54E − 08–4.83E − 03	208
Organismal development	3.30E − 08–4.83E − 03	260
Hematological system development and function	6.96E − 08–4.92E − 03	258
Immune cell trafficking	6.96E − 08–4.43E − 03	268
Embryonic development	8.16E − 07–3.34E − 03	147
Canonical pathway analysis/name	*P* value	Ratio
Antigen presentation pathway	1.89E − 05	12/42 (0.286)
Cytotoxic T lymphocyte-mediated apoptosis of target cells	1.35E − 04	12/88 (0.136)
Dendritic cell maturation	1.48E − 04	28/211 (0.133)
Molecular mechanisms of cancer	2.27E − 04	47/387 (0.121)
Crosstalk between dendritic cells and natural killer cells	2.7E − 04	18/106 (0.170)

### Clustering analysis of IPF and NSIP

**Fig. 1:**
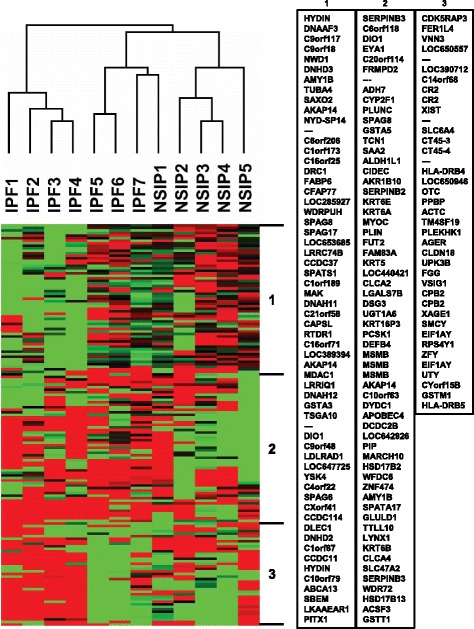
The dendrogram (top) shows similarities between samples, with shorter branches indicating a higher degree of similarity. The heat map (bottom) shows the expression level of 159 genes in each case. Red: upregulated genes; green: downregulated genes. In the right panel, the names of 159 genes are listed

An unsupervised two-dimensional hierarchical clustering algorithm was used to analyze similarities among samples and genes by using data obtained from expression profiles of 12 patients with IIP (Fig. 1). After filtering using the criteria described in materials and methods, 159 genes remained. As shown in the dendrogram, three major groups—IPF1–4, IPF5–7 and NSIP1, and NSIP2–5—were distinguishable based on expression data, suggesting that the transcriptional profiles of IPF and NSIP were similar (Fig. 1).

### Validation of gene array data with immunohistochemistry

**Fig. 2:**
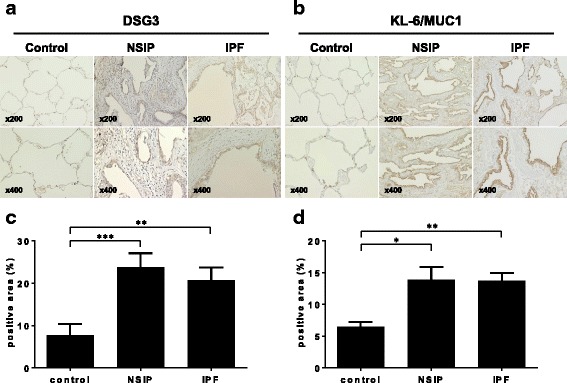
The expression of (**a**) DSG3 and (**b**) KL-6/MUC1 are strong in the lung affected by IPF or NSIP as compared to the controls. Morphometric analysis for (**c**) DSG3 and (**d**) KL-6/MUC1 confirmed that the rate of positively stained area is significantly high in the lung affected by IPF or NSIP as compared to the controls. *P < 0.05; **P < 0.01; ***P < 0.001

To validate the gene expression data at protein level, we selected two of the upregulated genes, *DSG3* and *KL-6/MUC1*, for immunohistochemical analysis. *DSG3* showed the highest upregulation in gene expression analysis, indicating its potential as a novel biomarker for IIPs. KL-6/MUC1 is well studied and is a clinically approved biomarker for IIPs [26, 30–33]. Clinical characteristics of the 10 patients with IIPs included in the immunohistochemical analysis were similar to those of the patients included in the microarray analysis (Table 1). As shown in Fig. 2a, DSG3 was mainly detected in the bronchiolar/alveolar epithelium and to a lesser extent in the fibrotic interstitium in IIP patients. The percentage of DSG3-positive areas in both IPF and NSIP lungs were significantly higher than those in control lungs (Fig. 2c). In agreement with earlier studies [26, 30, 33], KL-6/MUC1 was expressed by type II pneumocytes in all the lung specimens (Fig. 2b). Furthermore, continuous KL-6/MUC1 staining was observed on the cell surface of regenerating type II pneumocytes in IIP patients, in contrast with normal lung tissue in which a discontinuous pattern was observed (Fig. 2b). The percentage of KL-6/MUC1-positive areas in both IPF and NSIP lung were significantly higher than that in control lungs (Fig. 2d).

## Discussion

A genome-wide gene expression analysis revealed that several genes were up- or downregulated in the lung tissue of Japanese IIP patients compared to control subjects. Among them, *DSG3* showed the highest upregulation in IIP lung as compared to control lung, and was considered to be a potential novel biomarker for IIPs. Subsequently, the function and pathway analysis demonstrated that genes and pathways related to cancer, cell death and survival, and cellular growth and proliferation were differentially expressed in Japanese IIP patients. These results suggest the possibility that several molecules involved in cancer cell growth can be novel biomarkers that can potentially be used for diagnosis or serve as therapeutic targets for IIPs.

In total, 98 genes were upregulated in the lung tissue of Japanese IIP patients compared to control subjects. Among the upregulated genes, *DSG3* showed the highest upregulation indicating its potential as a novel biomarker for IIPs. DSG3, a member of desmoglein family, is a calcium-binding transmembrane glycoprotein component of desmosomes in epithelial cells [34]. Under normal conditions*,* DSG3 is expressed in oral mucosa and esophagus, but not in lungs [34]. Therefore, DSG3 expression in IIP lung may be due to the differences in cell adhesion properties between normal pneumocytes and regenerative pneumocytes in IIP lung. Our study shows for the first time that DSG3 expression is significantly different between IPF or NSIP lungs and the control lungs, suggesting a novel biomarker for the diagnosis of IIP. Actually, it has been reported that DSG3 can be the useful biomarker for squamous cell lung cancer [35]. As IIP and squamous cell lung cancer often occur simultaneously, both of these diseases may share the similar pathogenesis especially in the way cell adhesion properties are altered. In addition, some other molecules, which are known to be involved in cell-cell interaction and/or adhesion, were also shown to be upregulated. For example, KL-6/MUC1, which has been approved by Japan’s Health Insurance Program as a diagnostic marker for ILDs since 1999 and is currently in wide clinical use in Japan was upregulated in IIP lungs. Given that the key pathologic features of IIPs are considered to be epithelial cell damage and abnormal regeneration [36], we believe that our results from gene expression analysis are quite reasonable since these cell adhesion molecules are abundantly expressed in IIP tissues.

Other upregulated genes included transmembrane/secretory proteins such as DDR1, killer cell lectin-like receptor subfamily D member 1 (*KLRD1*) and toll-like receptor 10 (*TLR10*). These may also act as useful biomarkers since their cell surface localization makes them easily accessible to diagnostic methods and therapeutics. The biological and clinicopathologic significance of these candidate genes awaits validation through analysis of protein expression profiles in lung tissue obtained from IIP patients, as well as functional assays such as gene knockdown. Their potential as diagnostic markers in serum can be evaluated by enzyme-linked immunosorbent assay [37], but a possible caveat is that gene expression in lung tissue and serum levels of the gene product are not always correlated. More recently, a mass spectrometry-based technique, multiple reaction monitoring, has proven to be a useful method for detecting proteins without specific antibodies [38, 39]. Thus, a high-throughput serum proteome analysis using this system combined with microarray gene expression profiling would be ideal for selecting candidate serum biomarkers.

We also demonstrated that lot of genes are downregulated in the lung tissue of Japanese IIP patients (Table 3). Among them, *DEFA 1* and *3* are strongly downregulated; these genes are known to belong to human neutrophil peptides (HNPs) and the serum levels of HNPs have been reported to be elevated in patients with interstitial pneumonia associated with systemic sclerosis [40] and also in those with acute exacerbation of IIPs [41]. Our results would support the hypothesis that HNPs play important roles in the pathogenesis of IIPs. In addition, we also found that *IL-10*, which has been known as an inhibitor of cytokine production by Th1 cells is downregulated. As Th1 cytokine has been demonstrated to play important role in the progression of lung fibrosis [42], we can speculate that the downregulation of IL-10 accompanied with increased production of Th1 cytokine may strongly promote the fibrotic change in the lung.

Interestingly, IPA analysis in our study revealed that IIP had a profile that was more similar to cancer than to inflammatory responses and immunological diseases. These findings were in marked contrast to those in patients with chronic hypersensitivity pneumonitis (CHP); several pathways related to inflammatory responses and immunological diseases were differentially expressed in patients with CHP [43]. The canonical pathway analysis implicated dendritic cell maturation and molecular mechanisms of cancer pathways in IIP; indeed, the dendritic cell maturation signaling pathway is targeted by several molecular targeted agents mainly for chronic myelogenous leukemia such as nilotinib and dasatinib which inhibit Bcr-Abl tyrosine kinase activity [44, 45]. We speculate that these molecular targeted agents which interferes the dendritic cell maturation signaling pathway may also be beneficial for IIPs. Further study is required to determine whether these agents can, in fact, limit the progression of pulmonary fibrosis.

The differentially expressed genes and pathways in the patients with IIPs identified in the present study showed substantial overlap with those reported in the previous studies [17–19]. Selman M. et al. and Yang IV. et al. reported that genes encoding extracellular matrix molecules, cell surface molecules and cell adhesion molecules were highly expressed in IPF [17, 19]. In our study, *COL7A1*, which is involved in the extracellular matrix, *MUC1*, *KLRD1* and *TLR10*, which are the cell surface molecules, and *PCDHGA9* and *DSG3*, which are involved in cell-cell adhesion are upregulated in the patients with IIPs as compared to control. In addition, our results that functional pathways related to cellular growth and development are differentially expressed in the patients with IIPs as compared to control are similar to the results reported by Selman et al. [17]. Based on these results, we can speculate that the genes and pathways differentially expressed in the patients with IIPs are not much different between Japanese and Caucasians.

The transcriptional profiles of IPF and NSIP were similar and only minor differences in gene expression were identified, consistent with the results of several previous investigations [18, 19]. It is still possible that differences exist and may have been detected if multiple samples from different lobes of the lung had been separately analyzed, since lung disease by nature has a patchy distribution and also because IPF and NSIP may coexist in the same lung [46, 47]. Moreover, fibrotic NSIP in some patients has a presentation similar to IPF. The classification of IPF and fibrotic NSIP as separate diseases has recently been challenged, and it has been suggested that they share a common clinical phenotype and pathogenesis [48]. Importantly, patients with NSIP included in the present study were mostly consisted of fibrotic NSIP. The results presented here lend support to the reclassification of these IIP subtypes as a single clinical entity.

Although this study showed promising results, it has some limitations. First, control RNA for microarray analysis was derived from Caucasian subjects, because control RNA derived from Japanese subjects was not commercially available. Considering the ethnic differences in the relationship between genetic variants and the presence of IIP [49, 50], we cannot apply the results from the present study to Japanese patients with IIP without validation study. Second, the number of the subjects included in the immunohistochemical analysis is relatively small. We need further prospective studies with larger sample size in order to confirm the utility of DSG3 as the biomarker for IIPs.

## Conclusions

To summarize, the genome-wide gene expression analysis of Japanese IIP patients revealed a set of upregulated genes including *DSG3*, a promising novel biomarker for IIPs. The differentially expressed genes between IIP patients and controls are implicated in cancer, cell death and survival, and cellular growth and proliferation. This dataset provides a resource for future studies investigating the molecular mechanisms underlying the development and progression of pulmonary fibrosis as well as a collection of molecules that can be targeted by novel therapeutics.

## Abbreviations

CNTNAP3B: Contactin-associated protein-like 3B
COL7A1: Collagen type VII, alpha 1
DDR1: Discoidin domain-containing receptor 1
DSG3: desmoglein 3
GFI1B: Growth factor independent protein 1B
HRP: Horseradish peroxidase
IIP: Idiopathic interstitial pneumonia
ILD: Interstitial lung disease
IPF: Idiopathic pulmonary fibrosis
KL-6: Krebs von den lungen-6
KLRD1: Killer cell lectin-like receptor subfamily D member 1
MUC1: Mucin 1
NSIP: Nonspecific interstitial pneumonia
PCDHGA9: protocadherin gamma-A9
TLR10: Toll-like receptor 10
UIP: Usual interstitial pneumonia

## References

[1] Kinnula VL, Ishikawa N, Bergmann U, Ohlmeier S (2009). Proteomic approaches for studying human parenchymal lung diseases. Expert Rev Proteomics.

[2] Raghu G, Collard HR, Egan JJ, Martinez FJ, Behr J, Brown KK (2011). An official ATS/ERS/JRS/ALAT statement: idiopathic pulmonary fibrosis: evidence-based guidelines for diagnosis and management. Am J Respir Crit Care Med.

[3] Travis WD, Costabel U, Hansell DM, King TE, Lynch DA, Nicholson AG (2013). An official American Thoracic Society/European Respiratory Society statement: update of the international multidisciplinary classification of the idiopathic interstitial pneumonias. Am J Respir Crit Care Med.

[4] Flaherty KR, Andrei AC, King TE, Raghu G, Colby TV, Wells A (2007). Idiopathic interstitial pneumonia: do community and academic physicians agree on diagnosis?. Am J Respir Crit Care Med.

[5] Selman M, Pardo A (2006). Role of epithelial cells in idiopathic pulmonary fibrosis: from innocent targets to serial killers. Proc Am Thorac Soc.

[6] Bando M, Sugiyama Y, Azuma A, Ebina M, Taniguchi H, Taguchi Y (2015). A prospective survey of idiopathic interstitial pneumonias in a web registry in Japan. Respir Investig..

[7] Taniguchi H, Ebina M, Kondoh Y, Ogura T, Azuma A, Suga M (2010). Pirfenidone in idiopathic pulmonary fibrosis. Eur Respir J.

[8] Noble PW, Albera C, Bradford WZ, Costabel U, Glassberg MK, Kardatzke D (2011). Pirfenidone in patients with idiopathic pulmonary fibrosis (CAPACITY): two randomised trials. Lancet.

[9] King TE, Bradford WZ, Castro-Bernardini S, Fagan EA, Glaspole I, Glassberg MK (2014). A phase 3 trial of pirfenidone in patients with idiopathic pulmonary fibrosis. N Engl J Med.

[10] Ogura T, Azuma A, Inoue Y, Taniguchi H, Chida K, Bando M (2015). All-case post-marketing surveillance of 1371 patients treated with pirfenidone for idiopathic pulmonary fibrosis. Respir Investig.

[11] Richeldi L, du Bois RM, Raghu G, Azuma A, Brown KK, Costabel U (2014). Efficacy and safety of nintedanib in idiopathic pulmonary fibrosis. N Engl J Med.

[12] Kaminski N, Rosas IO (2006). Gene expression profiling as a window into idiopathic pulmonary fibrosis pathogenesis: can we identify the right target genes?. Proc Am Thorac Soc.

[13] Taniwaki M, Daigo Y, Ishikawa N, Takano A, Tsunoda T, Yasui W (2006). Gene expression profiles of small-cell lung cancers: molecular signatures of lung cancer. Int J Oncol.

[14] Ishikawa N, Daigo Y, Yasui W, Inai K, Nishimura H, Tsuchiya E (2004). ADAM8 as a novel serological and histochemical marker for lung cancer. Clin Cancer Res.

[15] Ishikawa N, Daigo Y, Takano A, Taniwaki M, Kato T, Hayama S (2005). Increases of amphiregulin and transforming growth factor-alpha in serum as predictors of poor response to gefitinib among patients with advanced non-small cell lung cancers. Cancer Res.

[16] Ishikawa N, Takano A, Yasui W, Inai K, Nishimura H, Ito H (2007). Cancer-testis antigen lymphocyte antigen 6 complex locus K is a serologic biomarker and a therapeutic target for lung and esophageal carcinomas. Cancer Res.

[17] Selman M, Pardo A, Barrera L, Estrada A, Watson SR, Wilson K (2006). Gene expression profiles distinguish idiopathic pulmonary fibrosis from hypersensitivity pneumonitis. Am J Respir Crit Care Med.

[18] Selman M, Carrillo G, Estrada A, Mejia M, Becerril C, Cisneros J (2007). Accelerated variant of idiopathic pulmonary fibrosis: clinical behavior and gene expression pattern. PLoS One.

[19] Yang IV, Burch LH, Steele MP, Savov JD, Hollingsworth JW, McElvania-Tekippe E (2007). Gene expression profiling of familial and sporadic interstitial pneumonia. Am J Respir Crit Care Med.

[20] Konishi K, Gibson KF, Lindell KO, Richards TJ, Zhang Y, Dhir R (2009). Gene expression profiles of acute exacerbations of idiopathic pulmonary fibrosis. Am J Respir Crit Care Med.

[21] Patel NM, Kawut SM, Jelic S, Arcasoy SM, Lederer DJ, Borczuk AC (2013). Pulmonary arteriole gene expression signature in idiopathic pulmonary fibrosis. Eur Respir J.

[22] American Thoracic Society, European Respiratory Society. American Thoracic Society/European Respiratory Society International Multidisciplinary Consensus Classification of the Idiopathic Interstitial Pneumonias. Am J Respir Crit Care Med. 2002;165: 277–304. 10.1164/ajrccm.165.2.ats01 11790668

[23] Page RD (1996). TreeView: an application to display phylogenetic trees on personal computers. Comput Appl Biosci.

[24] Eisen MB, Spellman PT, Brown PO, Botstein D (1998). Cluster analysis and display of genome-wide expression patterns. Proc Natl Acad Sci U S A.

[25] Tanaka S, Hattori N, Ishikawa N, Shoda H, Takano A, Nishino R (2012). Krebs von den Lungen-6 (KL-6) is a prognostic biomarker in patients with surgically resected nonsmall cell lung cancer. Int J Cancer.

[26] Kohno N, Akiyama M, Kyoizumi S, Hakoda M, Kobuke K, Yamakido M (1988). Detection of soluble tumor-associated antigens in sera and effusions using novel monoclonal antibodies, KL-3 and KL-6, against lung adenocarcinoma. Jpn J Clin Oncol.

[27] Ishikawa N, Ohlmeier S, Salmenkivi K, Myllärniemi M, Rahman I, Mazur W (2010). Hemoglobin α and β are ubiquitous in the human lung, decline in idiopathic pulmonary fibrosis but not in COPD. Respir Res.

[28] Dunn OJ (1964). Multiple comparisons using rank sums. Technometrics.

[29] Avivi-Green C, Singal M, Vogel WF (2006). Discoidin domain receptor 1-deficient mice are resistant to bleomycin-induced lung fibrosis. Am J Respir Crit Care Med.

[30] Kohno N, Kyoizumi S, Awaya Y, Fukuhara H, Yamakido M, Akiyama M (1989). New serum indicator of interstitial pneumonitis activity. Sialylated carbohydrate antigen KL-6. Chest.

[31] Ohnishi H, Yokoyama A, Kondo K, Hamada H, Abe M, Nishimura K (2002). Comparative study of KL-6, surfactant protein-a, surfactant protein-D, and monocyte chemoattractant protein-1 as serum markers for interstitial lung diseases. Am J Respir Crit Care Med.

[32] Ishikawa N, Hattori N, Yokoyama A, Kohno N (2012). Utility of KL-6/MUC1 in the clinical management of interstitial lung diseases. Respir Investig..

[33] Ohtsuki Y, Fujita J, Hachisuka Y, Uomoto M, Okada Y, Yoshinouchi T (2007). Immunohistochemical and immunoelectron microscopic studies of the localization of KL-6 and epithelial membrane antigen (EMA) in presumably normal pulmonary tissue and in interstitial pneumonia. Med Mol Morphol.

[34] Shirakata Y, Amagai M, Hanakawa Y, Nishikawa T, Hashimoto K (1998). Lack of mucosal involvement in pemphigus foliaceus may be due to low expression of desmoglein 1. J Invest Dermatol.

[35] Savci-Heijink CD, Kosari F, Aubry MC, Caron BL, Sun Z, Yang P (2009). The role of desmoglein-3 in the diagnosis of squamous cell carcinoma of the lung. Am J Pathol.

[36] Selman M, King TE, Pardo A (2001). American thoracic society; European Respiratory Society; American College of Chest Physicians. Idiopathic pulmonary fibrosis: prevailing and evolving hypotheses about its pathogenesis and implications for therapy. Ann Intern Med.

[37] Daigo Y, Nakamura Y (2008). From cancer genomics to thoracic oncology: discovery of new biomarkers and therapeutic targets for lung and esophageal carcinoma. Gen Thorac Cardiovasc Surg.

[38] Ishihara M, Araya N, Sato T, Tatsuguchi A, Saichi N, Utsunomiya A (2013). Preapoptotic protease calpain-2 is frequently suppressed in adult T-cell leukemia. Blood.

[39] Ueda K, Ishikawa N, Tatsuguchi A, Saichi N, Fujii R, Nakagawa H (2014). Antibody-coupled monolithic silica microtips for highthroughput molecular profiling of circulating exosomes. Sci Rep.

[40] Sakamoto N, Kakugawa T, Hara A, Nakashima S, Yura H, Harada T (2015). Association of elevated α-defensin levels with interstitial pneumonia in patients with systemic sclerosis. Respir Res.

[41] Sakamoto N, Ishimatsu Y, Kakugawa T, Yura H, Tomonaga M, Harada T (2015). Elevated plasma α-defensins in patients with acute exacerbation of fibrotic interstitial pneumonia. Respir Med.

[42] Burdick MD, Murray LA, Keane MP, Xue YY, Zisman DA, Belperio JA (2005). CXCL11 attenuates bleomycin-induced pulmonary fibrosis via inhibition of vascular remodeling. Am J Respir Crit Care Med.

[43] Horimasu Y, Ishikawa N, Iwamoto H, Ohshimo S, Hamada H, Hattori N, Okada M, Arihiro K, Ohtsuki Y, Kohno N (2017). Clinical and molecular features of rapidly progressive chronic hypersensitivity pneumonitis. Sarcoidosis Vasc Diffuse Lung Dis.

[44] Li J, Rix U, Fang B, Bai Y, Edwards A, Colinge J (2010). A chemical and phosphoproteomic haracterization of dasatinib action in lung cancer. Nat Chem Biol.

[45] Day E, Waters B, Spiegel K, Alnadaf T, Manley PW, Buchdunger E (2008). Inhibition of collagen-induced discoidin domain receptor 1 and 2 activation by imatinib, nilotinib and dasatinib. Eur J Pharmacol.

[46] Flaherty KR, Travis WD, Colby TV, Toews GB, Kazerooni EA, Gross BH (2001). Histopathologic variability in usual and nonspecific interstitial pneumonias. Am J Respir Crit Care Med.

[47] Monaghan H, Wells AU, Colby TV, du Bois RM, Hansell DM, Nicholson AG (2004). Prognostic implications of histologic patterns in multiple surgical lung biopsies from patients with idiopathic interstitial pneumonias. Chest.

[48] Maher TM, Wells AU, Laurent GJ (2007). Idiopathic pulmonary fibrosis: multiple causes and multiple mechanisms?. Eur Respir J.

[49] Horimasu Y, Ohshimo S, Bonella F, Tanaka S, Ishikawa N, Hattori N (2015). MUC5B promoter polymorphism in Japanese patients with idiopathic pulmonary fibrosis. Respirology.

[50] Peljto AL, Selman M, Kim DS, Murphy E, Tucker L, Pardo A (2015). The MUC5B promoter polymorphism is associated with idiopathic pulmonary fibrosis in a Mexican cohort but is rare among Asian ancestries. Chest.

